# European context of the diversity and phylogenetic position of SARS-CoV-2 sequences from Polish COVID-19 patients

**DOI:** 10.1007/s13353-020-00603-2

**Published:** 2021-01-05

**Authors:** Szymon Hryhorowicz, Adam Ustaszewski, Marta Kaczmarek-Ryś, Emilia Lis, Michał Witt, Andrzej Pławski, Ewa Ziętkiewicz

**Affiliations:** Institute of Human Genetics, Polish Academy of Sciences, 60-479 Poznan, Poland

**Keywords:** Coronavirus, Whole RNA genome sequencing, Haplotypes, Population, Phylogenetics, Epidemiology

## Abstract

To provide a comprehensive analysis of the SARS-CoV-2 sequence diversity in Poland in the European context. All publicly available (*n* = 115; GISAID database) whole-genome SARS-Cov-2 sequences from Polish samples, including those obtained during coronavirus testing performed in our COVID-19 Lab, were examined. Multiple sequence alignment of Polish isolates, phylogenetic analysis (ML tree), and multidimensional scaling (based on the pairwise DNA distances) were complemented by the comparison of the coronavirus clades frequency and diversity in the subset of over 5000 European GISAID sequences. Approximately seventy-seven percent of isolates in the European dataset carried frequent and ubiquitously found haplotypes; the remaining haplotype diversity was population-specific and resulted from population-specific mutations, homoplasies, and recombinations. Coronavirus strains circulating in Poland represented the variability found in other European countries. The prevalence of clades circulating in Poland was shifted in favor of GR, both in terms of the diversity (number of distinct haplotypes) and the frequency (number of isolates) of the clade. Polish-specific haplotypes were rare and could be explained by changes affecting common European strains. The analysis of the whole viral genomes allowed detection of several tight clusters of isolates, presumably reflecting local outbreaks. New mutations, homoplasies, and, to a smaller extent, recombinations increase SARS-CoV-2 haplotype diversity, but the majority of these variants do not increase in frequency and remains rare and population-specific. The spectrum of SARS-CoV-2 haplotypes in the Polish dataset reflects many independent transfers from a variety of sources, followed by many local outbreaks. The prevalence of the sequences belonging to the GR clade among Polish isolates is consistent with the European trend of the GR clade frequency increase.

## Introduction

The first reports of a new type of coronavirus-caused pneumonia, of then-unknown etiology, have been reported on 17/11/2019 in Wuhan, Hubei Province, China. The incidence of the disease has soon increased exponentially, moving to other regions of the world (Tang et al. 2020a). At the end of January 2020, COVID-19, the severe acute respiratory syndrome caused by the infection with coronavirus type 2, has been declared by the World Health Organization (WHO) a public health emergency of international importance. COVID-19 has taken on huge proportions, soon reaching a pandemic dimension. At the date of manuscript completing (5/9/2020), there were more than 27 million confirmed cases of the disease, with more than 880,000 deaths recorded worldwide (Coronavirus update (Live) n.d.); in Europe, over 3.7 million cases and 209,000 casualties were reported. In Poland, the first case of COVID-19 was diagnosed on 4/3/2020, in a man who returned from Germany (Thomson Reuters, Poland Reports First Coronavirus Case – Health Minister, March 4th, 2020). On March 6th, the Health Minister confirmed further four cases of COVID-19: a couple who came back from Italy, a man who returned from the UK, and a woman who traveled together with the zero patient. On 13/3/2020, the borders were closed, and social distancing implemented. Despite these precautions, infections spread throughout the country. As of 5/9/2020, 70,387 confirmed cases were reported in Poland, of which 2113 people have died.

SARS-CoV-2, which causes COVID-19, is a member of *Coronaviridae* family of enveloped, positive-sense RNA viruses type beta, which also encompasses SARS-CoV-1 and MERS-CoV (Harapan et al. 2020; Shaw et al. 2020; Feng et al. 2020). Like other viruses, SARS-CoV-2 constantly mutates, which results in the emergence of coronavirus subtypes (clades, subclades, haplotypes) (e.g., (Mercatelli and Giorgio 2020; Gudbjartsson et al. 2020; Hodcroft et al. 2020)). Coronavirus variability and evolution in different populations is crucial in the context of understanding its pathogenicity and proliferation dynamics. The full scale of the viral genome variability can best be appreciated when the whole viral genome sequence is analyzed. The unprecedented, ongoing global effort has resulted in the wealth of publicly available SARS-CoV-2 sequences deposited in the database hosted by GISAID, the Global Initiative on Sharing All Influenza Data (Elbe and Buckland-Merrett 2017; Shu and McCauley 2017). GISAID database is presently the most comprehensive, constantly updated repository of full-length SARS-CoV-2 sequences from all over the world (GISAID 2020).

Many papers concerning analyses of SARS-CoV-2 diversity in different countries or world regions have been published during the last few months, e.g., world (Mercatelli and Giorgio 2020; Yang et al. 2020), Europe and USA (Worobey et al. 2020), France (Gambaro et al. 2020), Germany (Walker et al. 2020), Italy (Licastro et al. 2020; Stefanelli et al. 2020), Iceland (Gudbjartsson et al. 2020), Croatia (Jurak et al. 2020), USA (Brufsky 2020), Chile (Rodriguez-Morales et al. 2020; Castillo et al. 2020), India (Somasundaram et al. 2020), Australia (Eden et al. 2020), and more focusing on other non-European populations. Slavic countries, including Poland, remain underrepresented in these studies, mostly since SARS-CoV-2 genomes have only recently become available.

To close this gap, we examined 115 whole-genome SARS-Cov-2 sequences from Polish samples, i.e., all currently available in the GISAID database, including those obtained during coronavirus testing performed in the COVID-19 Lab at the Institute of Human Genetics PAS. Polish coronavirus sequences were analyzed in the context of SARS-Cov-2 variability in other European countries observed until April 9th, four weeks after European border restrictions had been imposed.

## Materials and methods

### Diagnostic testing of COVID-19 patients in the Institute of Human Genetics

Four patients were from Wielkopolska region, and two from Opole and Lodz Voivodeships in Poland. All had a mild or asymptomatic course of the COVID-19 disease, and were quarantined. Samples for SARS-CoV-2 testing, taken according to WHO guidelines (WHO Team 2020), were nose and throat swabs, immersed in the R9F buffer (A&A Biotechnology, Gdynia, Poland). RNA extraction was performed using Viral DNA/RNA reagent kit (A&A Biotechnology, Gdynia, Poland). Real-time PCR was performed following the manufacturer’s protocol, using Gensig Coronavirus COVID-19 Real-Time PCR Assay (Primerdesign Ltd., York House, School Lane, Chandler’s Ford, UK), confirming that the samples were SARS-CoV-2-positive.

### SARS-CoV-2 RNA genome sequencing

Samples concentration was quantified using a fluorometric-based Qubit RNA HS Assay. All RNA samples were DNase-treated, and RNA integrity number was estimated (Agilent 2100 Bioanalyzer). SMART-Seq Stranded Kit (Takara) was used for library preparations, and whole transcriptome sequencing was performed using NovaSeq 6000 instrument (2 × 150 bp paired-end) (Illumina, San Diego, USA). Demultiplexing of the sequencing reads was performed with Illumina bcl2fastq (2.20). Adapters were trimmed with Skewer (v0.2.2) (Jiang et al. 2014). Trimmed raw reads were aligned to MN908947.3/NC_045512.2 using STAR (version 2.5.2b) (Dobin et al. 2013). The sets of aligned sequences (BAM files) were converted to FASTA format using samtools *mpileup* v1.9, bcftools *call* v1.7 (Li et al. 2009) and seqtk *seq* toolkit for processing sequences in FASTA/Q formats (Toolkit Seqtk n.d.).

The whole-genome RNA sequences of six SARS-CoV-2 isolates were deposited in GISAID (**EPI_ISL_450294**, **EPI_ISL_450295**, **EPI_ISL_450338**, **EPI_ISL_462480**, **EPI_ISL_485399**, **EPI_ISL_485400**) and GeneBank (**MT576645.1**, **MT499210.1**, **MT499208.1**, **MT499209.1 MT734046.1**, **MT734055.1**) databases.

### Selection of SARS-Cov-2 sequences from GISAID database

European (non-Polish) SARS-CoV-2 sequences from GISAID, used to define haplotypes, were selected as follows. Only high-coverage entries describing samples collected up to 9/4/2020 were considered. At the date of sequence download, a total of ~ 14,400 such sequences from thirty-five European countries were available from GISAID; at that time, ~ 780,000 cases had been reported in Europe (Coronavirus update (Live) n.d.). Many GISAID sequences were shorter than the 29,903 nucleotides (nt) reference, because of gaps at both ends; the analyzed sequences were trimmed (54 nt at 5′ and 103 nt at 3′), to obtain uniform length (29,746 nt). Sequences with longer end-gaps, and with internal stretches of Ns (unknown nucleotide in the sequence) longer than consecutive 6 nt, were excluded. As a result, ~ 5180 European sequences from twenty-seven countries were used for the initial sequence alignment. After manual inspection of the alignment, ~ 170 sequences with single Ns or ambiguous reads at the haplotype-defining positions were found and excluded from further comparisons, leaving 5013 isolates used for the analysis of haplotypes and clades diversity.

All Polish SARS-CoV-2 sequences (*n* = 115) available in GISAID were downloaded, without any restriction concerning dates of sample collection (58 samples collected between March and April 9th, and 59 between April 10th and mid-June); all but one (EPI_ISL_416488, Vero line) were from original isolates. Sequences used in the analyses were trimmed at both ends of the full-length viral sequence, as described for European sequences. However, internal positions with Ns, if not encompassing positions used to determine the clade affiliation, were conservatively replaced by the sequence of the general SARS-CoV-2 consensus. In this way, 115 Polish SARS-CoV-2 sequences were retained for the analysis, while the impact of sequencing errors or ambiguities on phylogenetic analyses was minimized.

### SARS-CoV-2 nomenclature

The GISAID-based nomenclature is based on marker mutations within six high-level phylogenetic groupings, according to Mercatelli and Giorgio (2020). A parallel school of SARS-CoV-2 nomenclature, with year-based clades A, B, and C, is used in the US-based ncov-NextStrain database (Gudbjartsson et al. 2020; Hodcroft et al. 2020). GISAID-based nomenclature was used throughout this work; however, references to NextStrain database names were also provided where possible (Table 1).

**Table 1:** Characterization and occurrence of sequence changes at haplotype-defining positions among 115 Polish isolates of SARS-CoV-2. Protein annotation and localization in the genome is based on Van Dorp et al. (2020); GISAID clades are as in Mercatelli and Giorgio (2020), NextStrain as in Gudbjartsson et al. (2020). “der,” derived

Nucleotide variant	Effect in protein	Localization in the genome	GISAID clades and haplogroups defined by the mutations	NextStrain Clades	Occurrence in Polish samples
C241T	-	5′UTR	G superclade	A2	71
C313T	p.L16L	Nsp1	GR-1	-	1
C1059T	p.T85I	Nsp2	GHI new clade	A2a2a	0
C1594T	p.S263S	Nsp2	GH-3	-	2
C2416T	p.Y537Y	Nsp2	GH-1	-	1
C3037T	p.F924F	Nsp3	G superclade	A2	71
C4002T	p.T428I	Nsp3	GR-3	-	10
G4255T	p.P512P	Nsp3	G-3-1	-	2
A8072G	p.N1785D	Nsp3	GR-9	-	12
C8782T	p.S76S	Nsp4	S clade	-	0
C9223T	p.H223H	Nsp4	V-der, G-der; homoplasic	-	1
T9477A	p.F308Y	Nsp4	S-2	-	1
G10097T	p.G15S	Nsp5	GR-3	A2a1b	10
G11083T	p.L37F	Nsp6	V clade; G-der; homoplasic	A1a, A3	3
C13536T	p.Y23Y	Nsp12	GR-3	-	10
C14408T	p.P4714L	Nsp12	G superclade	A2a	71
C14786T	p.A440V	Nsp12	GH-der; GR-der; homoplasic	-	1
C14805T	p.Y406Y	Nsp12	V-1 clade; S-der; homoplasic	A1a1	3
T17247C	p.P504L	Nsp13	V-1-1	A1a1a	1
A20268G	p.L216L	Nsp15	G-1	A2a3	1
G20578T	p.V320L	Nsp15	GH-1-2	-	1
A23403G	p.D614G	S gene	G superclade	A2	71
C23731T	p.T723T	S gene	GR-3	A2a1b	10
G24368T	p.D936Y	S gene	GHI-5	-	1
G24077T	p.D839Y	S gene	G-6	-	2
C25350T	p.P1263L	S gene	G-10	A2a10	2
G25429T	p.V13L	Orf3A	G-12	A2a7	1
G25563T	p.Q57H	Orf3A	GH and GHI clades	A2a2	0
G25979T	p.G136V	Orf3A	S-2-1-1	-	1
G26144T	p.G21V	Orf3A	V clade	A1a	0
A26530G	p.D3G	M gene	G-3	A2a5	2
C27046T	p.T175M	M gene	GRM	A2a1a	1
T28144C	p.L84S	Orf8	S clade	B	0
C28657T	p.D128D	N gene	S-der; G-der; GR-der; homoplasic	-	1
C28863T	p.S197L	N gene	S-2	-	1
GGG 28881-3 AAC	p.RG203-204KR	N gene	GR clade	A2a1	41
G29734C	-	3'UTR	G-1-1	-	1
Mutations rare in Europe (in less than 3% isolates)					
C106T	-	5′UTR	GR-der; homoplasic	-	12
C337T	p.R24R	Nsp1	GR-der; homoplasic	-	6
A2869G	p.V50V	Nsp3	GR-der	-	1
A3587G	p.H290Y	Nsp3	GR-der; only in Polish isolates	-	8
G5572A	p.M951I	Nsp3	GR-der; only in Polish isolates	-	9
A6133G	p.K1138K	Nsp3	GHI-der	-	4
G7936A	p.A1739A	Nsp3	GR-der; only in Polish isolates	-	8
G20419T	p.D267Y	Nsp15	GHI-der	-	5
C21707T	p.H49Y	S gene	L-der; G-der; GR-der; homoplasic	-	1

### Phylogenetic analysis

The whole-genome sequences of SARS-CoV-2 from Polish and European individuals were aligned using sequence identity algorithm MAFFT v 7.471 (Katoh and Standley 2013), using MN908947.3 as the reference sequence. Translation and gene distribution along the viral sequence were based on earlier annotations (Van Dorp et al. 2020). Phylogenetic relations between the Polish sequences were analyzed using the DNAML algorithm in Phylip v3.69 (Felsenstein 2005), with Tamura-Nei model and uniform rate of nucleotide substitution; the strength of the branches was assessed in Phylip v3.69 using bootstrap with 100 iterations. The diversity of Polish sequences was analyzed using DNADIST algorithm in Phylip v3.69, and their genetic distances were presented as multidimensional scaling (MDS) graph. In both DNAML and MDS analyses, the consensus clade sequences were included to indicate the position of Polish sequences within the main clades.

## Results

### Definition of European SARS-CoV-2 clades and haplotypes

European SARS-Cov-2 sequences from GISAID (5013, excluding Polish isolates) were analyzed to characterize the diversity of the six major clades: L, V, S, G, GH, GR. One major haplogroup within the G superclade, GHI defined by the presence of C1059T (p.T85I) on the background of GH, was granted a subclade status because of its high frequency (~ 15.5% isolates with the GHI consensus or derived sequences), advanced differentiation (twenty-eight derived haplotypes within the clade), and ubiquitous presence in many European populations. In fact, GHI overcomes its mother clade, GH, whose frequency and diversification are much lower (~ 5.5%, and twenty derived haplotypes).

Haplotypes within the clades were distinguished based on the shared presence of mutations along the SARS-CoV-2 genomic sequence; positions with mutations in fewer than 3% (16/5013) of the European set of sequences were not included in haplotype definition. Two hundred sixty-three haplotypes, including clade consensus and the derived sequences, were discerned based on the selection of 110 diagnostic positions. The haplotype definition reflected their clade affiliation (indicated by capital letter/s), followed by arbitrarily assigned numbers indicating haplotypes derived from the clade consensus by acquiring consecutive mutations; the presence of derived alleles at homoplasic sites was indicated by lowercase letters.

Twenty-three percent (61/263) of the European haplotype diversity (the number of extant haplotype variants, not to be confused with the frequency of these variants among the analyzed isolates) was represented by haplotypes, which were present in at least 3% isolates and found in at least three country populations; collectively, 77% (3844/5013) of all isolates in the analyzed European dataset carried these common variants. To find what mechanisms contributed to the occurrence of new variants comprising the remaining part of European diversity, we analyzed the structure of these, mostly population-specific, haplotypes. Only ~ 12% (32/263) of the haplotype diversity was due to the variants defined by new, apparently stable mutations on the background of frequent European haplotypes. These variants, found in 15% (771/5013) of the isolates, were either rare or present at elevated frequencies only in single populations, reflecting local outbreaks-related propagation of a given sequence. New variants caused by the presence of homoplasic mutations accounted for the largest part, 36%, of the European haplotype diversity. Almost 20 homoplasic sites were identified in the analyzed dataset; each of these mutations passed the criteria of the frequency > 3%, but the resulting haplotype variants were restricted to single populations or, in most cases, to single isolates (they collectively accounted for 6%, 297/5013 of the isolates). Another ~ 17% of the identified haplotypes (46/263) could be explained by back mutations, homoplasies, or, most parsimoniously, by recombination between the common haplotypes found at the high frequencies in the respective populations. Eleven of these possible recombinants were distinguished from their parental haplotypes at more than one haplotype-defining positions. Almost all of the purportedly recombined variants were restricted to one or few isolates, and collectively accounted for less than 2% (99/5013) of all the analyzed European isolates. The remaining 11% of the haplotypes (found in less than 1% of the isolates) could not be explained by single mutational events.

A more comprehensive analysis of the haplotype diversity in European SARS-CoV-2 samples is not presented in this study; the diversity of clades and haplotype definitions within each clade were used as a reference to characterize Polish SARS-CoV-2 sequences.

### Haplotypes in Polish SARS-CoV-2 isolates

The description of mutations, which determine clade/haplogroup affiliation of SARS-CoV-2 sequences in 115 Polish samples, is given in Table 1. The numbering of positions was based on the full-length (29,903 nt) MN908947.3 reference (L clade). Nine polymorphic positions, which did not fulfill the frequency criteria of haplotype-defining mutations within the European data set, were included in the analysis for the sake of a better characterization of Polish isolates (Table 1). Six of these positions (including three homoplasic) were present in less than 3% European isolates, and three were found only in Polish samples. Altogether, 52 diagnostic positions were included in the definition of haplotypes identified in the Polish dataset.

**Fig. 1:**
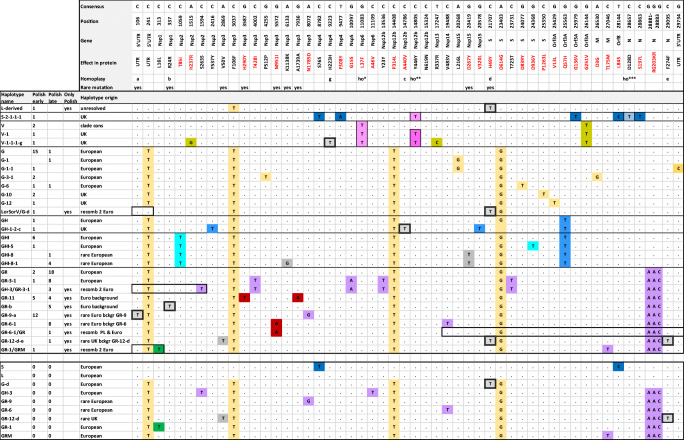
Schematic representation of the affiliation of Polish isolates with European SARS-Cov-2 clades and subclades. The number of sequences with a given haplotype is shown for the subset of isolates collected until and after April 9th (“early” and “late,” respectively). Haplotype origin column provides short information regarding the presence of each haplotype (or its background) among European isolates. Dots in the alignment indicate identity with the reference. Heavy boxes indicate minimal region of the presumed recombination events. Thin boxes at single positions indicate homoplasic mutations (based on the analysis of the reference cohort of ~ 5013 European isolates). G11083T (ho*), C14805T (ho**), and C28657T (ho***) differ from other homoplasic sites in that their presence in some haplotypes is stable; the respective haplotypes are defined by the numbers. Other homoplasies (double-boxed) are found at a low frequency on different backgrounds (based on the analysis of European data; not shown); their presence in haplotype is indicated by lowercase letters. Colors of the diagnostic positions are kept throughout each clade; rare mutations are shown in gray; Polish-specific mutations are in dark-red. A lower panel, with haplotype variants not found in Polish dataset, is presented to explain rare haplotype background or putative parental haplotypes involved in possible recombination events

The schematic diagram, presenting Polish haplotypes, is shown in Fig. 1. All main European clades were represented. The majority of the 30 haplotypes found in Polish isolates were observed at high frequencies in at least three other European countries from the reference cohort of twenty-seven countries. Ten Polish-specific haplotypes were observed. Three were due to the otherwise absent or very rare mutations (GR-11, GR-6-1, and the derived GR-6-1/GR, GR-12-d-e) on either frequent or rare European background. Other Polish-specific haplotypes were due to mutations at sites characterized by homoplasy in the European dataset (GR-b, GR-9-a) or appeared to be a result of recombination between the common variants (GR-1/GRM, GH-3/GR-3-1, LorV/G-d, L/G/L-d). Alternatively, one of the putatively recombined haplotypes could be explained by reverse mutations (positions 241, 3037, and 19,488) or homoplasies at sites that are not extensively homoplasic in Europe (positions 313, 1594).

### Polish SARS-CoV-2 diversity in the context of non-Polish European data

The majority of Polish samples belonged to the G superclade: GR constituted 58%, G—23%, and GH/GHI—12%. Sequences classified as belonging to the L/V/S clades accounted for only 6% of the analyzed Polish isolates.

**Fig. 2:**
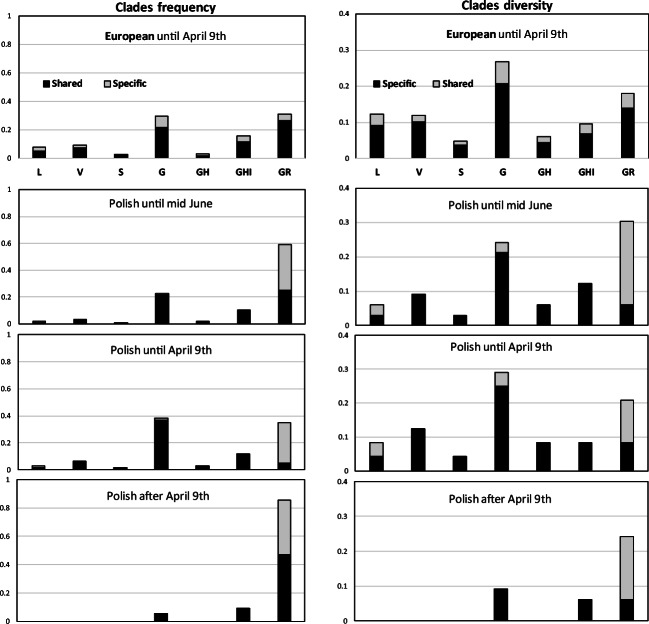
SARS-CoV-2 clades frequency and diversity in Poland and Europe. Left panels: Clades frequency (the proportion of isolates falling into each clade) in European and Polish samples. Right panels: Clades diversity (the proportion of distinct haplotypes within each clade). The darker shading indicates frequency and diversity of haplotypes shared among different countries, the lighter depicts sequences, whose occurrence is restricted to single countries. (A) European samples; (B–D) Polish samples; collection dates are indicated on each graph

Comparison of the clades frequency (the number of isolates within each clade) and their diversity (the number of distinct derived haplotypes within each clade) in Polish and European datasets indicated the scarcity of Polish-specific haplotypes in clades G, GH/GHI, and L/V/S clades (Fig. 2A, B). This was especially evident in the G clade, which in Europe was characterized by the highest ratio of population-specific haplotypes. In contrast, the increased frequency of Polish isolates within the GR clade was accompanied by the 4-fold excess of Polish-specific haplotypes, in stark contrast to the European samples, where country-specific haplotypes contributed only 1/3 to this clade diversity. However, when we separately characterized subsets of Polish isolates collected until April 9th (Fig. 2C) and after that date (Fig. 2D), the prevalence of the GR clade was seen only in the later subset, while the clades distribution in earlier Polish sequences was more consistent with that seen in the European dataset collected during the same period.

### Phylogenetic position and genetic diversity of Polish SARS-CoV-2 samples

**Fig. 3:**
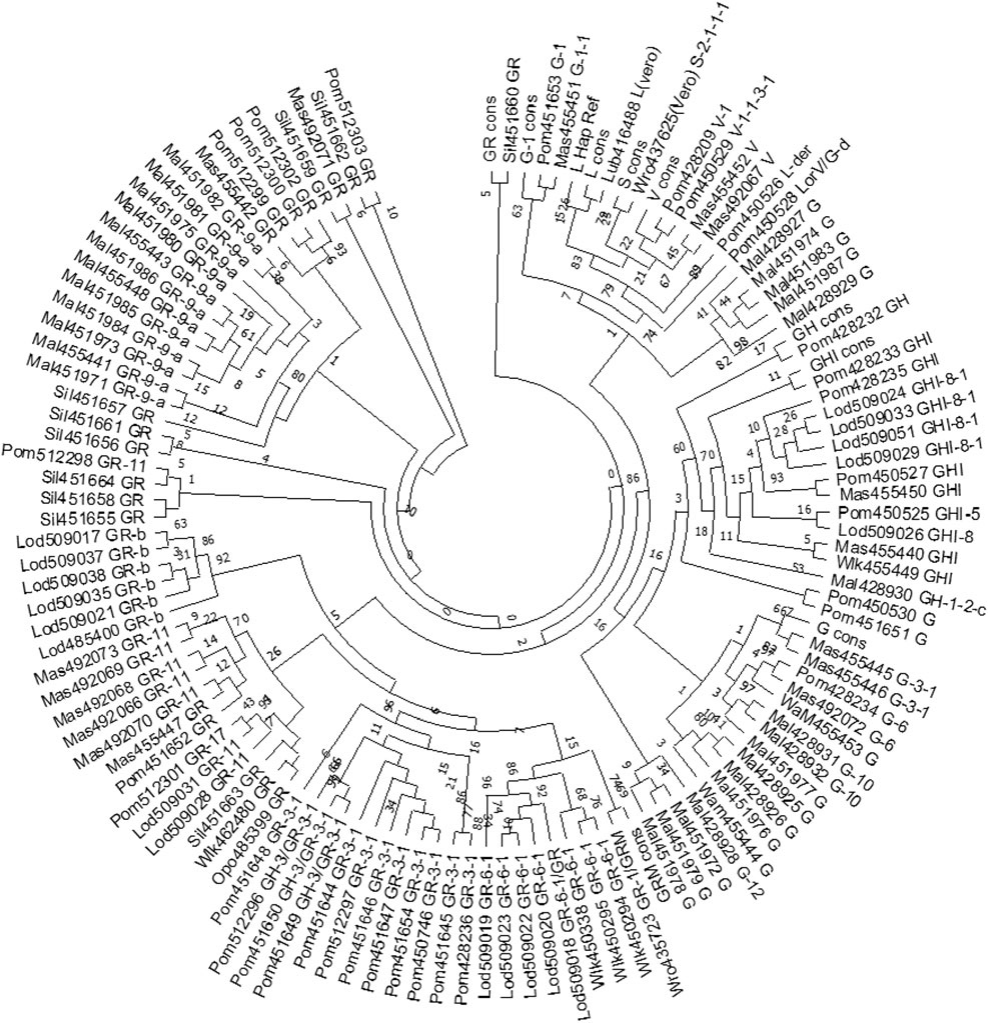
Max likelihood tree of Polish SARS-CoV-2 sequences. Each leaf contains the abbreviated regional affiliation of the isolate, GISAID number, and the haplotype name based on the analysis of haplotype-defining positions; consensus sequences of the main clades are also shown. Please note that this is an unrooted tree. The three-letter code describes regional origin of the samples, described in detail in Fig. 3; haplotype names shown next to each isolate number correspond are as in Fig. 1

Positions of Polish SARS-CoV-2 isolates within the maximum likelihood tree, shown in Fig. 3, compared to the consensus sequences of the major clades, confirmed their affiliation as assessed from the inspection of the sequences.

**Fig. 4:**
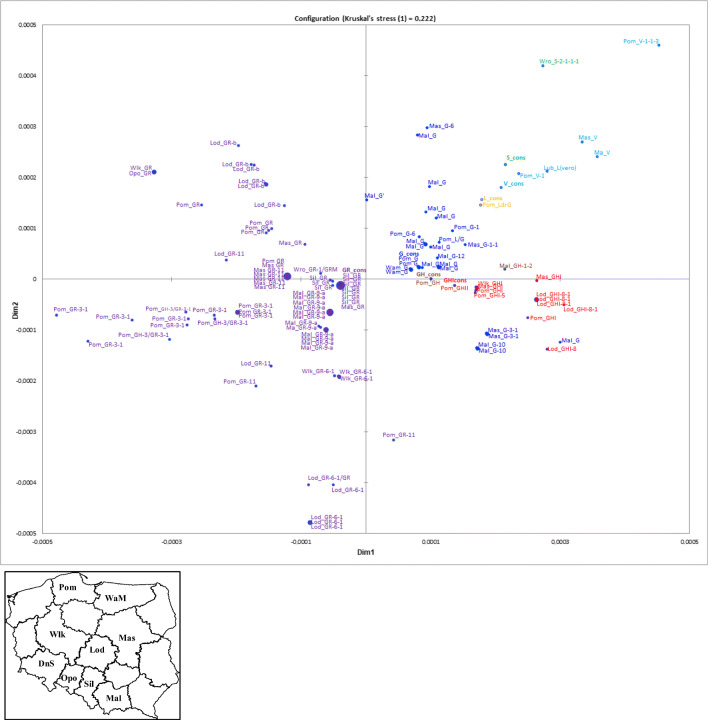
Multidimensional scaling reflecting genetic distances between sequences of Polish SARS-CoV-2 isolates. Different colors indicate SARS-CoV-2 clade affiliation of the isolates; the positions of clades consensus are indicated. Because of the extremely small/minimal genetic distance separating isolates, some points overlap; to indicate this, the clusters are revealed by a single marker of larger diameter. The three-letter code describes regional origin of the samples. WaM, Warmia-Mazury (*n* = 2); Wlk, Wielkopolska (*n* = 5); Mal, Malopolska (*n* = 28); Wro, Wroclaw (Dolnoslaskie) (*n* = 2); Opo, Opole (*n* = 1); Sil, Silesia (*n* = 10); Mas, Masovia (*n* = 16); Lod, Lodz (*n* = 1); Pom, Pomerania (*n* = 24); see inset for the localization on the map of Poland

The multidimensional scaling (MDS) analysis (Fig. 4), based on the pairwise DNA distance matrix, illustrated the genetic divergence of Polish isolates. The tight clustering of some of the sequences from the GR clade (especially GR-9-a haplotypes in samples from Malopolska, GR-11 from Masovia, and some GR from Silesia) indicated their small (if any) divergence and pointed to separate local outbreak events.

## Discussion

The analysis of European and of Polish coronavirus sequences confirmed that the SARS-CoV-2 evolution is relatively stable. The number of derived haplotypes due to new mutations observed on single haplotype backgrounds was moderate, and isolates carrying such haplotypes were usually restricted to single populations (exemplified by G20419T in haplotypes GHI-8). This is consistent with the previous reports that coronaviruses change more slowly than most other RNA viruses, probably because of the “proofreading” activity of Nsp12 exonuclease; SARS-Cov-2 mutation rate underlying global diversity has been estimated at ~ 6 × 10^−4^ nucleotides/genome/year (Van Dorp et al. 2020).

A relatively large part of the population-specific haplotype diversity resulted from homoplasies. Homoplasic mutations are commonly found in the SARS-CoV-2 genome (Van Dorp et al. 2020; De Maio et al. 2020). Many homoplasies resemble hot-spot mutations—new alleles are found on a large variety of haplotypes, and their presence does not contribute to the stable evolution of the sequence. On the other hand, some of the homoplasic mutations remain stably associated with the specific haplotype background and can be used to trace sequence evolution. For example, C14805T (designed ho* in Fig. 1) was found on two different, but stable backgrounds—one in the S and another in the V clade. G11083T (ho**), with well-established homoplasic character (Van Dorp et al. 2020), was stably associated with the V clade, and highly recurrent among European sequences from other clades; it was not observed in any of the Polish sequences from the G superclade. The homoplasic character of other mutations in Polish haplotypes (e.g., those indicated by lowercase letters a and b in haplotype names in Fig. 1) was inferred from the analysis of reference European dataset; their stable association with single haplotypes in Polish isolates most probably reflected founder effects resulting from local outbreaks of SARS-CoV-2 carrying these mutations.

Recombination of two sequences appeared to be the most parsimonious explanation for the structure of some Polish-specific haplotypes, e.g., GH-3/GR-3-1; GR-1/GRM; LorV/G-d; GR-6-1/GR (see Fig. 1). Inspection of the haplotype structure in over 5000 European isolates revealed that up to 17% of all the variants could be explained by recombination. While some of these variants may reflect convergent evolution (involving recurrent mutations, back mutations) (Wertheim 2020), or even sequencing errors, recombination remains a plausible scenario, especially when more than one polymorphic sites are involved (as was the case of 11 haplotypes) and when the alleged mother variants are present at high frequency in the same populations. The active recombination of SARS-CoV-2 has been reported and discussed in several studies (Yi 2020; De Maio et al. 2020; Nie et al. 2020; VanInsberghe et al. 2020; Varabyou et al. 2020; Wertheim 2020). While the detailed results were not concordant, all the studies underscored the rare occurrence of recombinants. It has been suggested that the actual rate of recombination might be higher, but not detectable due to the low diversity of the SARS-CoV-2 sequences (VanInsberghe et al. 2020; Wertheim 2020). Interestingly, Koelle’s group (VanInsberghe et al. 2020), who did not confirm recombinants reported by Yi (2020), has reported five other recombinants in the analysis of 47,390 sequences grouped in 14 clades defined by 37 positions. In our study, the search for recombinants was based on the analysis of 263 haplotypes defined by 110 positions; the higher resolution could explain why more purported recombinants were revealed. It is worth mentioning that ~ 1.6% of the European sequences analyzed by us (80 isolates not included among 5013 used for the haplotype diversity analysis) were characterized by heteroplasmy, seen as ambiguous readouts at sites involved in clades or haplotypes definition (e.g., c.28881-3 on the G background, suggesting coinfection with GR; c.25350 on the G background, suggesting coinfection with G-11). While the possibility of sequencing error or contamination cannot be excluded, the presence of heteroplasmy in SARS-CoV-2, also reported in other studies (Tang et al. 2020b), implies double infection events, and speaks in favor of the possible role of recombination in the emergence of some haplotype variants. While, based on our data, it cannot be excluded that a part of the existing SARS-CoV-2 diversity is due to recent recombines events, the rare occurrence of isolates carrying the purportedly recombined variants suggests that these sequences did not proliferate extensively, consistent with the previous reports (VanInsberghe et al. 2020; Wertheim 2020). More data, and perhaps longer time, given the slow evolution of SARS-CoV-2 sequence, is needed to assess to what extent does recombination contribute to SARS-CoV-2 evolution (Wertheim 2020). Finally, while recombination is believed to underlie evolutionary jumps, which allow viruses to change their hosts (Su et al. 2016; Luk et al. 2019), the role of the present knowledge does not allow to assess whether recombination plays any role in SARS-CoV-2 acquiring specificity for human ACE2 receptor (Boni et al. 2020).

The frequency of certain haplotypes in different populations may change rapidly due to founder effects caused by local outbreaks, and this usually does not invoke selective advantage of such strains. While unsupervised assumption that the prevalence of any given SARS-CoV-2 strain indicates its increased virulence should be avoided, examples of the global spread of some coronavirus mutations deserve attention. The global increase of the G superclade frequency at the cost of S/V/L lineages, also seen in the Polish dataset, has led to the conclusion that the hallmark G superclade mutation, p.D614G substitution in the spike protein (A23403G), might be responsible for the increased virulence of the coronavirus (Brufsky 2020; Korber et al. 2020). The possible selective advantage of p.D614G facilitating interaction with the receptor on the surface of human cells is presently considered a plausible, albeit still not fully proven scenario (Zhang et al. 2020; Korber et al. 2020; Plante et al. 2020; Volz et al. 2020; Grubaugh et al. 2020).

The analysis of all currently available full-length SARS-CoV-2 sequences (*n* = 115) from Polish isolates revealed that most of the haplotypes seen in the analyzed set are also found at varying frequencies in other European countries (Fig. 1). Coronavirus strains, which circulate in Poland, appear therefore to originate in many independent transfers from various populations. This is consistent with the fact that the epidemic outbreak in France, Italy, Germany, UK, Finland, Belgium, and Sweden (Coronavirus update (Live) n.d.) preceded that in Poland by over two weeks, during which border restrictions were not yet imposed. By the time COVID-19 struck Poland, all major coronavirus clades were already present in Europe (Mercatelli and Giorgio 2020; Worobey et al. 2020; Yang et al. 2020; Mavian et al. 2020a; Pachetti et al. 2020). With no rigorous epidemiological interview (history of travel, contacts of infected individuals, etc.), it is impossible to state what was the country of origin for particular transmission cases.

Similar to the reference European dataset, the majority of the analyzed Polish isolates belonged to the G superclade, encompassing clades G, GH/GHI, and GR. Sequences representing the older SARS-CoV-2 lineages (L, V, and S) were sparse among Polish samples. The relative frequency and diversity of the G and GH/GHI clades in Polish data were comparable with that in the rest of Europe. The scarcity of Polish-specific haplotypes in these clades suggested that almost all isolates observed in the analyzed dataset represent direct transfers from other European countries, which did not result in extensive local transmissions, similar to early coronavirus introductions in France (Gambaro et al. 2020). In contrast, the frequency of the GR clade in Polish samples (60%), much higher than observed in the European dataset (30%), revealed a scenario consistent with the successful expansion of this clade in Poland (Fig. 2A, B). In addition, the GR clade was more diversified than in the rest of Europe, in terms of the proportion of different haplotypes. The discrepancy in the GR clade abundance between the set of 115 Polish sequences collected between March and mid-June, and the European reference sequences collected until April 9th was mostly due to the contribution of Polish sequences collected after April 9th; the frequency of Polish isolates collected until April 9th was much closer to that in the European dataset. Indeed, the recent study on the SARS-CoV-2 geographical and temporal distribution in Europe, encompassing the period from January to mid-June (Alm et al. 2020), has indicated that the frequency of the GR clade in April was ~ 30%, consistent with our calculations based on the manual analysis of the GISAID data. However, early in June, the GR clade frequency in Europe overtook that of the other clades, and since then is on the constant rise. In mid-June, the overall European frequency of the GR clade exceeded 50% (Alm et al. 2020), which is much closer to ~ 60% calculated for 115 Polish sequences collected until that date, and to ~ 50% reported in Alm’s paper for the subset of 79 Polish isolates. Overall, these observations suggest that the changes in the frequency of SARS-CoV-2 clades in Poland follow the trend consistent with that observed in the rest of Europe.

The detailed analysis of the whole SARS-CoV-2 genome allowed identification of population-specific low-frequency mutations, which defined new haplotypes and indicated the common origin of groups of isolates. Furthermore, the analysis of haplotype divergence due to the accumulation of mutations at sites not used for haplotypes definition provided clues regarding their independent history. In the DNA distance–based MDS analysis (Fig. 3 and Fig. 4), some of the Polish sequences carrying the same haplotypes formed tight clusters, apparently reflecting local COVID-19 outbreaks (e.g., samples carrying Polish-specific GR-9-a or GR-11 haplotypes), while others (e.g., carrying frequent European GR-3-1 or G haplotypes) were randomly spread, presumably representing independent transfers from a variety of sources. Similar clustering was revealed in the DNAML tree. It has to be emphasized that the phylogenetic tree was only presented to show clustering of some sequences. Given that the Polish set of isolates represented an incomplete and biased fraction of coronavirus cases in Poland, no root was assigned to the phylogenetic tree; no phylogenetic inferences were made, to avoid overinterpretation of the data (Mavian et al. 2020b).

Fairly complete knowledge of the genetic diversity of SARS-CoV-2 is important for medical epidemiology, diagnostics, and prevention (Mavian et al. 2020a). Assigning virus isolates to main European clades is the foundation for such efforts, but only the whole RNA genome sequencing allows the detection of population-specific mutations and haplotypes. While they may have little value for reconstructing the SARS-CoV-2 evolution on the trans/continental scale, they are essential for the attempts to explain local pathways of virus spread and to identify undocumented local sources of COVID-19 outbreaks. Furthermore, recognizing the local prevalence of specific haplotypes may have a substantial impact on the accuracy of population-specific diagnostic tests.

Understanding the SARS-CoV-2 genomic variability is of particular importance for designing therapies or vaccines (Van Dorp et al. 2020; Weissmann et al. 2020), as it allows selection of evolutionarily constrained regions of the coronavirus genome, which should be preferentially targeted to avoid rapid drug and vaccine escape mutants. Here again, information on the variability of strains circulating in a given population will help to adjust future medical interventions to the population-specific profile of infections.

Our study has obvious limitations related to the small number of whole-genome sequences from Poland available in GISAID. The present estimate of infected people in the country with the population of more than 37.8 million exceeds 70 thousand, and the actual number may be much higher. To alleviate this problem, extensive testing of the whole population should be implemented. Importantly, once SARS-CoV-2 infections become identified, the representative sets of sequences should be obtained, including those from asymptomatic cases; this will be the first step towards understanding relations between the SARS-CoV-2 genetic subtype and its virulence and severity of the disease course.
